# Serotype epidemiology and multidrug resistance patterns of *Salmonellaenterica* infecting humans in Italy

**DOI:** 10.1186/s13099-016-0110-8

**Published:** 2016-06-01

**Authors:** Ilaria Frasson, Sabrina Bettanello, Ettore De Canale, Sara N. Richter, Giorgio Palù

**Affiliations:** Department of Molecular Medicine, University of Padua, via Gabelli 63, 35121 Padua, Italy; Microbiology and Virology Unit, Padua Teaching Hospital, via Giustiniani 2, 35121 Padua, Italy

**Keywords:** *Salmonella*, Multidrug resistance, Humans, Epidemiology

## Abstract

**Background:**

*Salmonella enterica* is the zoonotic agent most frequently responsible for foodborne infections in humans worldwide. In this work the presence of *S. enterica* was investigated in 734 unique enteropathogenic isolates collected from human patients between 2011 and 2012.

**Results:**

All *Salmonella* spp. isolates were subjected to serotyping and antimicrobial susceptibility testing. Isolates displaying phenotypes and antimicrobial susceptibility profiles different from the reference strains were genotipically characterized. Several plasmid-embedded resistance determinants were identified and characterized. Non-typhoidal serotypes were most frequently diagnosed; *monophasicSalmonellatyphimurium 1,4 [5],12:i*- and *S. typhimurium* represented the most prevalent serovars. Five isolates displayed phenotypes with extremely reduced susceptibility to antimicrobials: we detected multidrug resistance elements belonging to Ambler class A and class C in two non-typhoidal *S. enterica* serovars, i.e. *Rissen* and *monophasic S. typhimurium 1,4 [5],12:i*-, and in one typhoidal serovar, i.e., *Paratyphi B*. These resistance determinants have been so far almost exclusively associated with non-*Salmonella* members of the *Enterobacteriaceae* family. Alarmingly, two colistin resistant *Salmonella enteritidis* were also found.

**Conclusions:**

This work draws the attention to the still low, but rising, percentage of multidrug resistant *Salmonella* isolates infecting humans in Italy. Our results suggest that prompt monitoring of *Salmonella* serovar dissemination and resistance to antimicrobials is highly required.

## Background

*Salmonella enterica* (*S. enterica*) consists of more than 2600 different serovars, which, based on different antigens, are subgrouped into typhoidal and non-typhoidal *Salmonella* (NTS) serovars. These serotypes vary greatly in their virulence, natural reservoirs and susceptibility to antimicrobials [1]. Typhoidal *S. enterica* serotypes *Typhi* and *Paratyphi A* and *B* infect humans as their exclusive reservoirs, causing outbreaks of life-threatening typhoid fever [2]. Outbreaks of typhoid fever are mainly restricted to low-income countries, while they represent less than 1 % infections in the developed countries. In contrast, non-typhoid salmonellosis usually gives rise to acute but self-resolving gastroenteritis, with no need of antimicrobial therapy; life-threatening invasive infections may occur in vulnerable patients [3].

In 2015, the European food and safety authority (EFSA) reported that the investment of the European Union in *Salmonella* control measures is yielding noticeable results, with only 20.4 confirmed cases per 100,000 population. Nevertheless, non-typhoidal salmonellosis remains the major cause of foodborne zoonotic outbreaks [4].

The increasing number of antibiotic-resistant *Salmonella* spp. against first line antimicrobial agents (aminopenicillins, trimethorpim-sulfametoxazole and chloramphenicol) intensified the empirical use of fluoroquinolones and third generation cephalosporins [5–7]. Noticeably, these classes of antibiotics have been recommended for the treatment of *Salmonella* infections in animals [8]. In the last years, retail food, livestock and companion animals have been the main sources of NTS transmission [9, 10].

Genetic determinants conferring resistance to beta-lactams and cephalosporins embedded in widely spreading plasmids have been extensively characterized in other *Enterobacteriaceae*, such as *E. coli* and *K. pneumoniae*. The detailed genetic analysis of circulating plasmids helped containing MDR outbreaks [11, 12]. Thus, monitoring serovar distribution and antimicrobial resistance of *S. enterica* in humans may be important in controlling the infection.

The Microbiology and Virology Unit of the Padua Teaching Hospital, National Reference Centre for enteropathogenic bacteria and antimicrobial resistance epidemiology for the Northeast Italy, has begun in 2009 a strict surveillance of circulating non-pansusceptible *Enterobacteriaceae,* among which *Salmonella* spp.

Here we report the phenotypic and genotypic analysis of *Salmonella* isolates infecting human patients between January 2011 and December 2012 in Northeast Italy, analyzed at the Microbiology and Virology Unit of the Padua Teaching Hospital. We diagnosed and characterized multidrug resistance elements in two non-typhoidal *S. enterica* serovars, *Rissen* and *monophasictyphimurium*, and in one typhoidal serovar, *Paratyphi B*. The resistance determinants were embedded in plasmids with the ability to transfer between bacterial species. Two *S. enterica* serotype *Enteritidis* samples were characterized by colistin resistance.

## Methods

### Characterization of *Salmonella* strains: serotyping and antimicrobial susceptibility testing

A total of 734 unique enteropathogenic isolates, obtained exclusively from human patients, were received and characterized by the Microbiology and Virology Unit of the Padua Teaching Hospital. Samples were collected between January 2011 and December 2012, according to the current version of the Declaration of Helsinki. Putative *Salmonella* containing samples were plated on selective xylose lysine deoxycholate agar (XLD agar, Becton–Dickinson Italia, Milan, Italy) and colonies from pure cultures were characterized using a VITEK^®^ 2 automated system (bioMérieux, Marcy l’Étoile, France). The isolates identified as *Salmonella* spp. were subjected to serotyping by slide agglutination, according to the Kaufmann-white classification [13].

Antimicrobial susceptibility testing was performed on all confirmed *Salmonella* isolates, by the disc diffusion (Kirby-Bauer) method according to EUCAST criteria [14]. The following antibiotics (μg/disk) were tested on Mueller–Hinton agar (Difco Laboratories, Detroit, MI), according to Enter-Net specifications: nalidixic acid (30), ampicillin (10), cefotaxime (30), ciprofloxacin (5), chloramphenicol (30), gentamicin (10), kanamycin (30), streptomycin (10), sulphonamide (300), tetracycline (30), trimethoprim (5), ampicillin-clavulanic acid (20-10), cefalotin (30) and sulfamethoxazole-trimethoprim (23.75–1.25) (BD BB™ Sensi-Disk™, Becton–Dickinson Italia, Milan, Italy). Moreover, the disk diffusion test was performed to detect Extended-spectrum beta-lactamases (ESBL) (BD BB™ Sensi-Disk™, Becton–Dickinson Italia, Milan, Italy).

Colistin susceptibility was determined using Sensititre ARIS^®^ 2X (Trek Diagnostic Systems, Thermo Scientific Inc., Milan, Italy). *Escherichia coli* ATCC25922 and *E. coli* ATCC35218 were used as control strains.

### Identification of ESBL and AmpC determinants: plasmid transfer by transformation and conjugation experiments

Plasmid DNA was obtained from pure bacterial cultures performing phenol/chloroform extraction. Resistance genes (bla_TEM_, bla_SHV_, bla_CTX-M_, and AmpC) were detected by PCR as previously described [15, 16]. Positive PCR products were sequenced in an ABI3130xl sequencer (Applied Biosystems, Thermo Scientific Inc., Milan, Italy) and sequences were aligned to those present in GenBank using BLAST (http://blast.ncbi.nlm.nih.gov/Blast.cgi).

Plasmids containing PCR-confirmed resistance determinants were introduced into chemo-competent *E. coli* cells (SCSI, Agilent Technologies Italia, Milan, Italy). Transformants were plated on selective Mueller–Hinton (cefotaxime 2 mg/L or ampicillin 100 mg/L) (Becton–Dickinson & Co., DifcoTM) agar plates. The presence of plasmids was confirmed by PCR targeting the whole sequence of the *bla*_TEM/CTX/CMY_ gene; PCR products were treated with exosap and fully sequenced (ABI3130xl sequencer, Applied Biosystems). Microbial identification and antimicrobial susceptibility testing on transformants were performed at a VITEK^®^ two automated system (bioMérieux, Marcy l’Étoile, France).

For conjugation experiments, one colony of each donor (*S. enterica* serovars: *Rissen*, *monophasic S. typhimurium 1,4 [5],12:i*- and *Paratyphi B*) and the recipient strain *E. coli* J53 (F− met pro Azi^r^ Col^r^) were cultured separately under weak agitation in LB broth at 37 °C. Conjugation was performed incubating different dilutions (from 1:100 to 1:10) of donor suspensions with overnight culture of the recipient strain under low agitation. Mating assays were carried out at room temperature from 1 h to overnight depending on the donor strain. The transconjugants were selected on agar plates containing colistin (8 mg/L) and cefotaxime (2 mg/L) or ampicillin (100 mg/L). Microbial identification and antimicrobial susceptibility testing on transformants and transconjugants were performed by VITEK^®^ two automated system (bioMérieux, Marcy l’Étoile, France).

### Plasmid PCR-based replicon typing (PBRT)

All plasmids were analyzed by PCR-based replicon typing (PBRT) targeting the replicons of the major plasmid families occurring in *Enterobacteriaceae* [17]. Each vector was assigned to a specific Inc group. Plasmid typing was also performed in the obtained transformants and transconjugants.

### Amplification and analysis of pmrA and pmrB genes

Bacterial total DNA was obtained by heat shock from pure culture suspension. *PmrA* and *pmrB* genes, the major regulators of LPS modifications in *S. enterica*, were amplified as previously described [18]. PCR amplicons were sequenced by end-primers in an ABI3130xl sequencer (Applied Biosystems, Thermo Scientific Inc., Milan, Italy) and the obtained sequences were compared to the corresponding sequences in the NCBI database (http://www.ncbi.nlm.nih.gov/nucleotide).

## Results

### *Salmonella enterica* serotype distribution and antimicrobial susceptibility profiles

The screening of 734 enteropathogenic human specimens yielded identification of 350 *S. enterica* isolates. On the basis of the Kauffmann-White serotyping, three samples were positive for typhoidal *Salmonella* strains: two isolates were typed as *Salmonellatyphi*, whereas one belonged to the *Paratyphi B* serovar. The remaining 99 % samples belonged to different non-typhoidal serovars (NTS). The most common serotypes in the North-eastern part of Italy were *monophasic S. typhimurium 1,4 [5],12:i*- and *S. typhimurium*, which were retrieved in approximately 29.5 and 26.5 % samples, respectively (Table 1). The remaining samples belonged predominantly to *S. enteritidis*, *S. panama* and S. *napoli*; other NTS were detected to a minor extent (Table 1).

**Table 1:** Serotype ditribution of *Salmonella* *enterica* isolates diagnosed in this study

Serotype	% isolates/year
*S. typhimurium, monophasic 1,4 [5],,12:i*-	29.5
*S. typhimurium*	26.6
*S. enteritidis*	9.4
*S. panama*	5.5
*S. napoli*	3.2
*S. muenchen*	1.9
*S. derby*	1.6
*S. thomson*	1.2
*S. rissen*	1.2
*S. infantis*	1
Others	18
Not determined	0.9

Antimicrobial susceptibility testing showed that 38.4 % of isolates were susceptible to all classes of antibiotics. The two *Thyphi* serovar isolates belonged to this fully susceptible group. The remaining *Salmonella* isolates displayed different antimicrobial resistance profiles: (i) 12.1 % exhibited reduced susceptibility or resistance to two classes of antimicrobials; (ii) 13.4 % were classified as multidrug-resistant (MDR), i.e. they showed resistance against three or more non-structurally related antimicrobials; (iii) 36.1 % were resistant to ampicillin, streptomycin, sulphonamides and tetracycline (ASSuT resistant pattern). The resistance rate to quinolones and fluoroquinolones (i.e., nalidixic acid and ciprofloxacin) and third generation cephalosporins (i.e., cefotaxime, ceftazidime and ceftriaxone) was relatively low among the isolates. Fluoroquinolones and third generation cephalosporins are the antibiotics most widely used in the treatment of both human and animal infections. In detail, the isolates resistant to nalidixic acid and/or ciprofloxacin were 6 and 0.5 %, respectively. Reduced susceptibility to third generation cephalosporins was detected in 0.9 % isolates. Notably, 0.5 % isolates displayed a resistance phenotype against fourth generation cephalosporins (i.e., cefepime) as well. As reported in other countries, all isolates were fully susceptible to imipenem and meropenem [19, 20].

The presence of extended spectrum beta lactamases (ESBL) and acquired class C cephalosporinases (AmpC) was also investigated by double disk synergy assays. Three isolates displayed an ESBL/AmpC phenotype. These were further characterized at the molecular level to detect plasmid-borne resistance determinants and additional resistance mechanisms.

### Molecular analysis of resistance determinants of ESBL/AmpC producing strains

The three isolates that possessed an ESBL/AmpC phenotype belonged to the following serovars: *S. paratyphi B*, *S.* r*issen* and *monophasic S. typhimurium* 1,4 [5],12:i-. Characterization of resistance determinants was performed on total bacterial DNA extracted as previously described [16, 21]. The major resistance determinants, i.e., *bla*_TEM_, *bla*_SHV_, plasmidic AmpC genes and *bla*_CTX-M-type_, were amplified and sequenced. Genotypic analysis indicated that *S. paratyphi B* harbored *bla*_TEM-52_, *S. rissen* was positive for *bla*_CMY-2_, while *monophasicS.typhimurium* 1,4 [5],12:i- encoded *bla*_CTX-M-15_.

The detected molecular determinants belonged to Ambler Class A and C, which have been previously reported within mobile elements in other members of the *Enterobacteriaceae* family, responsible for human outbreaks [22]. Therefore, the possible spreading in *Salmonella* of these resistance determinants through highly diffusible plasmid was investigated.

Plasmid content of the three isolates was analyzed by multiple techniques. Plasmid DNA extractions were analyzed by PCR: the efficient amplification of the selected resistance genes indicated that all determinants conferred plasmid-mediated resistance. For epidemiological tracing purposes, backbones were characterized by PCR amplification of incompatibility groups to determine plasmid replicon typing (PBRT) [17]. This analysis indicated that *bla*_CMY-2_ was embedded in an IncA/C plasmid and *bla*_TEM-52_ in an IncI1-Iγ plasmid. Plasmid analysis of the *bla*_CTX-M-15_-positive *monophasic S. typhimurium* 1,4 [5],12:i- isolate displayed a double replicon profile, i.e. IncFIIs and IncFIA, indicating the presence of more than one vector or of a backbone with a multi-replicon status.

To confirm plasmid-embedding of the resistance determinants, IncI and IncF plasmids were transformed into *E. coli* and transformants were analyzed for replicon typing and for the presence of bla_CTX-M-15_ and bla_TEM-52_. Transformation experiments demonstrated that bla_CTX-M-15_ was contained in the IncFIA backbone, while no transformation of the IncFIIs plasmid and bla_CMY-2_ determinant was obtained. Plasmid size or degradation might account for the absence of transformants.

Many antibiotic resistance plasmids can promote horizontal dissemination of genetic traits among bacteria of different genera through the conjugation process [11]. For this reason, transferability of the resistance determinants was assessed by mating experiments. All three plasmids conjugated successfully: PBRT, along with the presence of the three resistance determinants, was confirmed by PCR in the recipient *E. coli J53* strain. The straightforward horizontal transfer of the three backbones suggested that both clonal spread and horizontal plasmid transfer may have contributed to *Salmonella* resistance dissemination in *Salmonella* isolates (Table 2).

**Table 2:** Characteristics of ESBL or AmpC-positive *Salmonella* isolates and transformed or transconjugated *E. coli* isolates analyzed in this study

Serovar	Antimicrobial susceptibility testing						ASSuT	PCR	PBRT
	AMX (mg/L)	CFPM (mg/L)	CAZ (mg/L)	CTX (mg/L)	FOX (diameter zone)	Additional resistances			
*S. rissen*	>8	≤1	>4	≥2	7 mm	GEN, STR, STX, TET	Positive	bla_CMY-2_	IncA/C
*monophasic S. typhimurium (1,4,[5],12:i:*-*)*	>8	≥4	>4	≥2	26 mm	KAN, GEN, STR, STX, TET, TMP	Positive	bla_CTX-M-15_	IncFIA, IncFIIS
*S. Paratyphi B*	>8	≥4	>4	≥2	26 mm	nd	Negative	bla_TEM-52_	I1-Ig
*E. coli* transformed bla_CTX-M-15_	>8	≥4	>4	≥2	–	nd	nd	bla_CTX-M-15_	IncFIA
*E. coli* transformed bla_TEM-52_	>8	≥4	>4	≥2	–	nd	nd	bla_TEM-52_	I1-Ig
*E. coli J53* bla_CMY-2_	>8	≤1	>4	≥2	–	nd	nd	bla_CMY-2_	IncA/C
*E. coli J53* bla_CTX-M-15_	>8	≥4	>4	≥2	–	nd	nd	bla_CTX-M-15_	IncFIA
*E. coli J53* bla_TEM-52_	>8	≥4	>4	≥2	–	nd	nd	bla_TEM-52_	I1-Ig

*AMX* amoxicillin; *CFPM* cefepime; *CAZ* ceftazidime; *CTX* cefotaxime; *FOX* cefoxitin; *KAN* kanamycin; *GEN* gentamicin; *STR* streptomycin; *SMX* sulfamethoxazole; *TET* tetracycline; *TMP* trimethoprim; *nd * not detected

### Colistin susceptibility testing

Because of the recent uprising rate of colistin resistance reported in *Enterobacteriaceae* [23, 24], susceptibility to colistin was also evaluated in all *Salmonella* isolates. Indeed, two isolates of *S. enterica* serotype *Enteritidis*, displayed MIC_90_ higher than 4 μg/mL and were thus considered resistant according to EUCAST breakpoints [14]. As mutations in the *pmrAB* operon were demonstrated to be involved in *Salmonella* resistance to colistin and other polymixins [18], *pmrA* and *pmrB* genes were amplified and sequenced. *PmrA* and *pmrB* nucleotide sequences were identical to those reported for colistin-susceptible isolates: no polymorphic positions with synonymic amino acid substitution, or point nucleotide mutation were detected, suggesting the presence of other resistance mechanisms responsible for the observed reduced susceptibility to colistin [25].

## Discussion

Non-typhoidal salmonellosis has not been hitherto considered a major public health risk in developed countries, such as the European Union member states. Up to date, only few epidemiology studies on *Salmonella* isolates infecting humans has been published so far [19, 26, 27].

In the present study, we presented the Italian epidemiology of *Salmonella* isolates infecting humans between 2011 and 2012, both in terms of serovar distribution and in terms of antimicrobial resistance patterns.

Our investigation highlighted that pathogenic *Salmonella* counted for the majority of all enteropathogenic pathogens (47.8 %) diagnosed by our laboratory, National Reference Centre for Enteropathogenic Bacteria for the Northeast Italy. In the 2-year period of the study, more than 11 different serotypes were identified: *monophasic S.typhimurium 1,4 [5],12:i:*- and *S. typhimurium* were the prominent circulating serovars, retrieved in almost 30 and 27 % of all samples, respectively. In contrast, the EFSA report on circulating zoonotic agents in 2013 indicated that *S. enteritidis* was the most prevalent *Salmonella* serovar detected in human samples [28].

Our epidemiological data are supported by recent works demonstrating that *S.typhimurium* and *monophasic S. typhimurium* 1,4 [5],12:i- have extremely high survival rates in the environment [29], can infect a broad range of species [30] and have enhanced their ability to cause salmonellosis in humans [19]. Moreover, these were the most common serotypes diagnosed in serious foodborne salmonellosis outbreaks in both humans and animals in 2012 [31]. Thus, *S.typhimurium* and *monophasic S. typhimurium* 1,4 [5],12:i- have to be considered emerging predominant serotypes in humans all around Europe.

In addition, our study pointed out that 61.6 % of analyzed *Salmonella* isolates were resistant to at least one antibiotic, with 13.3 % of samples classified as multidrug resistant. In accordance with our data, EFSA reported increasing multidrug resistance in *Salmonella* infecting humans, where *monophasic S. typhimurium* 1,4 [5],12:i- serotype exhibited the highest rate of multidrug resistance at the European level [32].

Despite the relatively low resistance rate against the clinically important third generation cephalosporins, molecular analysis of strains displaying ESBL/AmpC-producing phenotypes proved that the isolates bore worrisome plasmid-embedded resistance determinants, able to move through bacterial species: AmpC, such as CMY-2, and ESBL, such as TEM-52 and CTX-M-15. The three determinants have been more commonly associated with other members of the *Enterobacteriaceae* family [33]. Notably, the identified genes have been recently reported in human outbreaks of salmonellosis all around the world [19, 20, 34], associated with other *Salmonella* serovars and embedded in plasmids distinct from the ones traced in this study.

So far, ESBL have been rarely described in *S. paratyphi B* at the European level and have been never reported at the Italian level [35, 36]. Moreover, *Salmonella* serotype *Rissen* is a widespread contaminant of pigs and pork products [37] and up to date the presence of CMY-like cephalosporinases in this serovar had been reported only in South Korea in 2002 [38, 39]. Our data highlight the possible future spreading of resistance to first line antimicrobials through this serovar.

An additional worrisome feature described here is the detection of two isolates of *S. enteritidis* displaying resistance to colistin. In Europe, *S. enteritidis* is reported to be one of the most frequent serotypes diagnosed both in animals and in humans. No other studies detected and characterized, up to date, colistin-resistant isolates belonging to this serovar. Up to 2013, the highest proportions of resistance among *S. enteritidis* isolates were observed for nalidixic acid (19.5 %) and ampicillin (11.0 %), while resistance to third generation cephalosporins was generally detected at low levels [32]. The spreading of colistin-resistant *S. enteritidis* could worsen the clinical treatment of life-threatening cases of salmonellosis.

## Conclusions

Our results provide an outline of serotype distribution and rising antibiotic resistance among *S*. *enterica* causing salmonellosis in humans in Italy. The serotype landscape differs from the one presented by the EFSA reports and deserves to be further monitored.

The described plasmid-embedded resistance determinants circulating in *Salmonella* serovars should be closely surveilled, as they could possibly become a public health threat, as occurred in the last years for others *Enterobacteriaceae*, such as *K. pneuomoniae* [12, 22, 24, 33].

## Abbreviations

*S. enterica*: *Salmonella enterica*
NTS: non-typhoidal *Salmonella*
EFSA: European food and safety authority
*E. coli*: *Escherichia coli*
*K. pneumoniae*: *Klebsiella pneumoniae*
Azi^r^ Col^r^: sodium azide resistant and colistin resistant
ESBL: extended-spectrum beta-lactamases
PBRT: PCR-based replicon typing
MDR: multidrug resistant
ASSuT: ampicillin, streptomycin, sulfonamides, and tetracycline
